# Optimizing fitness: plastic flowering time in variable environments

**DOI:** 10.3389/fpls.2025.1690015

**Published:** 2025-10-07

**Authors:** Wei Dong, Yalin Zhang, Yue Xing, Kexin Chang, Nianwei Qiu, Yuguang Song

**Affiliations:** College of Life Sciences, Qufu Normal University, Qufu, China

**Keywords:** stress resistance, flowering time, phytohormone, environmental stress, regulation mechanism

## Abstract

Flowering time is a critical determinant of crop yield and adaptability, regulated by the integration of environmental cues, phytohormones, and genetic networks. Abiotic stresses such as drought, waterlogging, salinity, and high temperature, together with biotic stresses including pathogens, viruses, and herbivores, profoundly reshape flowering phenology through microRNAs, transcription factors, epigenetic modifications, and hormonal crosstalk. Phytohormones, especially ABA and GA, act as regulatory hubs coordinating stress adaptation and floral transition, though their effects vary across species and conditions. This review synthesizes recent advances in stress-mediated flowering regulation and emphasizes the challenges of balancing stress tolerance with yield stability. We propose that integrating multi-omics data, regulatory network modeling, and artificial intelligence will accelerate the breeding of stress-resilient cultivars with stable productivity.

## 1 Introduction

Recent abrupt environmental shifts have forced plants to undergo significant evolutionary adaptations to ensure survival. To mitigate the impacts of both internal and external environmental fluctuations on growth and reproduction, plants have developed sophisticated mechanisms to sense and respond to external cues such as light, temperature, and stress. Among these adaptive strategies, the regulation of flowering time plays a central role in coping with environmental challenges (Gu et al., 2022) and serves as a critical determinant of agricultural productivity (Ying et al., 2023).

Flowering is controlled by the integration of exogenous signals—including temperature, photoperiod and endogenous cues such as developmental stage, phytohormone levels, and nutrient status (Zhao et al., 2023). These signals converge on key floral integrators, including FLOWERING LOCUS T (FT) and SUPPRESSOR OF OVEREXPRESSION OF CONSTANS 1 (SOC1). The integrated signals are subsequently transmitted to the shoot apical meristem, where they activate floral identity genes such as *LEAFY* (*LFY*) and *APETALA1* (*AP1*), thereby precisely triggering the floral transition (Winter et al., 2015; Yu et al., 2016) (**Table 1**). This review systematically examines the molecular mechanisms through which plants and crops integrate light, temperature, phytohormones, and environmental stresses to fine-tune flowering. By doing so, we establish a comprehensive framework for understanding adaptive flowering networks under climatic perturbations and provide a conceptual basis for targeted flowering regulation and crop improvement strategies.

**Table 1:** The functions of flowering regulatory factor in plants.

Gene name	Molecular function	Biological function	Ref.
*CO*	Bind to FT promoter, and ABA and GI signaling regulate it	Early flowering	(Kinmonth-Schultz et al., 2021)
*FT*	Florigen activated by CO transcription	Early flowering	(Riboni et al., 2013) (Riboni et al., 2016)
*SOC1*	Mediate signals such as photoperiod and temperature, and act as a member of the MADS-box to regulate flowering	Early flowering	(Hwang et al., 2019)
*LFY*	Active the expression of floral organ characteristic genes by binding to downstream gene promoters	Early flowering	(Winter et al., 2015)
*AP1*	Active floral organ characteristic genes	Early flowering	(Yu et al., 2016)
*PIF4*	Promotes FT expression	Early flowering	(Koini et al., 2009)
*GI*	Regulation of CO recruitment to FT promoter	Early flowering	(Robustelli Test et al., 2025)
*SVP*	Suppressing transcription of flowering genes	Late flowering	(Wang et al., 2018) (Riboni et al., 2013)
*SPLs*	regulate flowering by the miR156/SPLs module	Early flowering	(Feng et al., 2021)
*GmamiR172c*	Promotes *LYF* and *FT* expression	Early flowering	(Li et al., 2016)
*HvumiRNA173b-5p*	Promotes TPS expression	Late flowering	(Swida-Barteczka et al., 2023)
*AtNAC79*	Unknown	Early flowering	(Sanjari et al., 2019)
*SiMYBS3*	Unknown	Early flowering	(Liu et al., 2023)
*HcCNGC27*	Promotes *FLC* expression. Represses *FT* and *SOC1* expression	Late flowering	(Chen et al., 2025)
*NTL8*	Represses *FT* expression	Late flowering	(Kim et al., 2007)
*OsELF3-1* *OsLUX*	Represses GI expression	Late flowering	(Andrade et al., 2022)
*EIN3* *EIL*	Mediate ethylene signaling, thereby activating the expression of the AP2/ERF family and inhibiting the expression of FT	Late flowering	(Guo and Ecker, 2004)
*GA20ox1* *GA3ox1*	Up-regulate the expression of bioactive GA and promote the expression of flowering promoters through the GA regulatory network	Early flowering	(Stavang et al., 2009) (Yamaguchi, 2008)
*BZR1*	Binding to the PIF4 promoter, it induces the expression of downstream BRs, thereby promoting *FT* transcription	Early flowering	(Ibañez et al., 2018)
*ABI3* *ABI4* *ABI5*	Promotes *FLC* expression	Late flowering	(Shu et al., 2018) (Wang et al., 2013) (Xu et al., 2022)

## 2 Abiotic stress-mediated regulation of flowering

**Figure 1:**
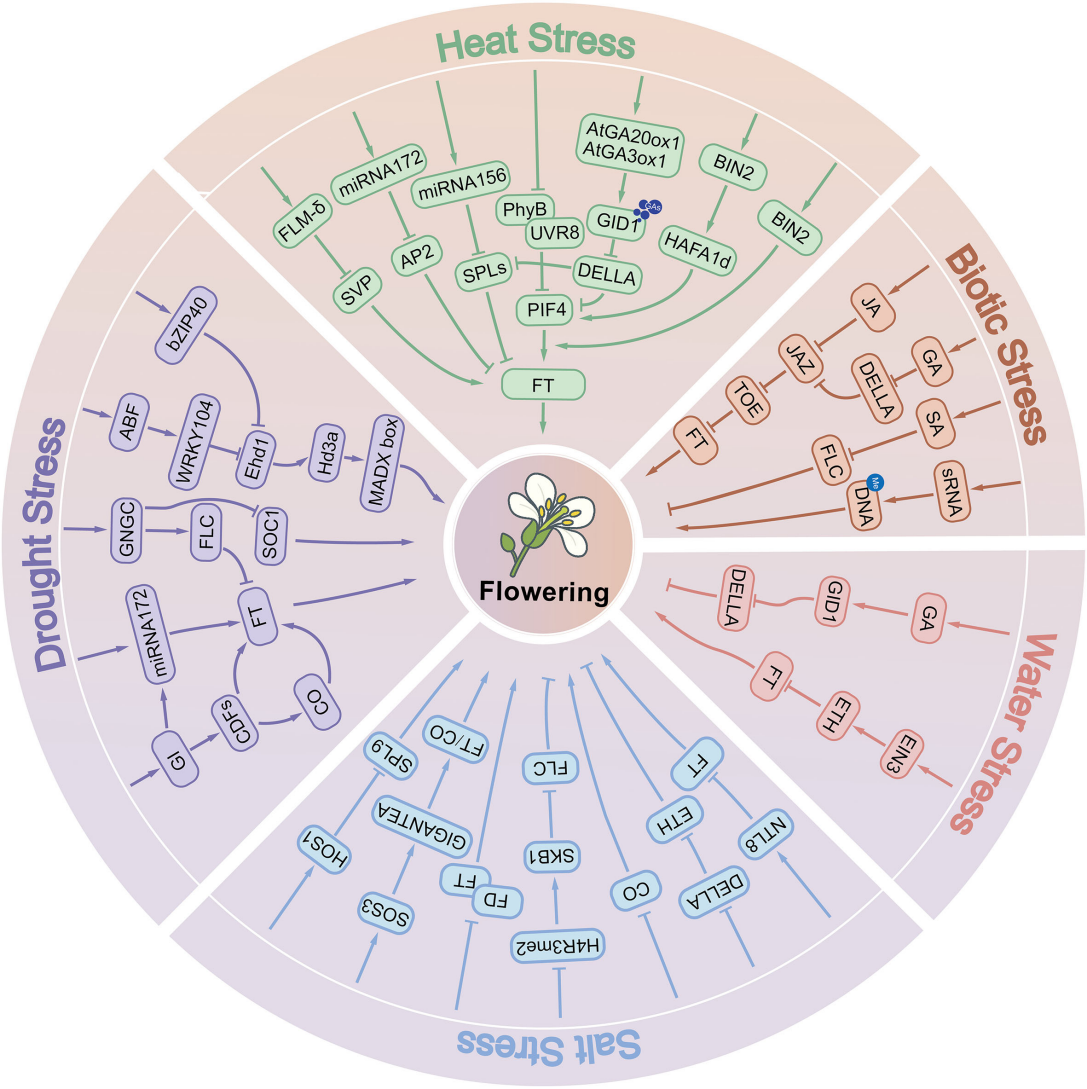
Molecular mechanisms underlying abiotic and biotic stress-mediated regulation of flowering time in plants.

Crop yield is highly sensitive to environmental conditions. The strategic adjustment of flowering phenology under stress represents a core adaptive mechanism for balancing reproductive fitness across generations while enhancing crop resilience. Within this regulatory framework, diverse biotic and abiotic stressors coordinately modulate flowering timing and stress tolerance through integrative signaling networks that converge on key developmental regulators (**Figure 1**).

### 2.1 Drought stress and flowering regulation

MicroRNAs (miRNAs) play crucial roles in mediating crop responses to drought stress. For example, in soybean, drought stress and abscisic acid (ABA) signaling synergistically induce gma-miR172c, which enhances ABA sensitivity by transcriptionally activating ABI3 and ABI5 (Li et al., 2016). This regulatory cascade promotes the accumulation of the flowering integrator FT, thereby accelerating floral transition under water-deficit conditions (**Figure 1**). A similar mechanism has been reported in barley, where drought-induced downregulation of miRNA173b-5p elevates trehalose accumulation. The metabolic intermediate trehalose-6-phosphate (T6P) subsequently promotes flowering via the trehalose biosynthetic pathway (Swida-Barteczka et al., 2023).

In parallel, transcription factors (TFs) play central roles in coordinating drought-mediated flowering. The NAC family, one of the largest TF families in crops, functions as a key regulator at the intersection of flowering time control and stress tolerance (Sanjari et al., 2019). For instance, overexpression of *NAC79* in *Arabidopsis* confers both drought tolerance and an early-flowering phenotype (Guo et al., 2017). Similarly, MYB TFs are widely involved in abiotic stress responses (Ai et al., 2023), with *MYBS3* overexpression shown to enhance drought tolerance while simultaneously promoting early flowering (Liu et al., 2023). By contrast, CNGC family genes exhibit an opposing effect: they enhance drought resistance through reactive oxygen species (ROS) scavenging and stress-gene activation, but delay floral transition by repressing *FT* and *SOC1* (Chen et al., 2025) (**Figure 1**). This highlights the complex regulatory trade-offs between stress adaptation and reproductive timing under drought conditions.

### 2.2 Waterlogging stress and flowering regulation

Crop responses to waterlogging stress represent a complex physiological process, with root hypoxia acting as the primary stress factor. Ethylene, a key gaseous phytohormone, functions not only as a central stress signal but also as a regulator of flowering, influencing floral timing through multilayered signaling networks (Guo and Ecker, 2004). At the molecular level, ethylene signaling is mediated by EIN3/EIL transcription factors, which activate AP2/ERF family proteins such as *ERF1*, while simultaneously repressing the flowering integrator *FT*, ultimately leading to delayed flowering (**Figure 1**). This ethylene-dependent regulation is tightly coupled with gibberellin (GA) metabolism and DELLA protein stability (**Figure 1**). Reduced GA levels inhibit DELLA degradation, resulting in DELLA accumulation and suppression of floral initiation (Achard et al., 2007). Furthermore, under combined waterlogging and shading stress in maize (Zea mays), ethylene overaccumulation interferes with flowering through a dual mechanism: repression of *FT* expression and reduced activity of the CONSTANS (CO) protein, both of which markedly delay the floral transition (Zhou et al., 2021). A comprehensive understanding of how waterlogging stress interacts with flowering pathways is essential for breeding cultivars with enhanced tolerance to both drought and waterlogging, thereby minimizing yield penalties under adverse environmental conditions.

### 2.3 Salt stress and flowering regulation

Salt stress represents a major abiotic constraint compromising crop growth, yield, and quality. As a leading contributor to global crop losses, salinity profoundly regulates flowering phenology through identified molecular pathways, providing foundational insights into the mechanistic basis of floral transition under salt stress. With respect to specific regulatory mechanisms, multiple molecular pathways and related proteins play key roles in salt stress-induced flowering delay in *Arabidopsis*. The plant-specific transcription factor NTL8 represses *FT* expression under high salinity (Kim et al., 2007), while salt stress concurrently suppresses *CO* transcription (Li et al., 2007) (**Figure 1**). Additionally, molecules related to floral initiation are closely involved in salt stress-regulated flowering. The floral initiator Shk1 kinase-binding protein 1 (SKB1) and symmetric dimethylation of histone H4 arginine 3 (H4R3sme2) play important roles in regulating flowering time under salt stress (**Figure 1**). Under salt stress, H4R3sme2 levels decrease, leading to the dissociation of SKB1 from chromatin, which in turn induces the expression of *FLOWERING LOCUS C* (*FLC*), ultimately delaying flowering (Zhang et al., 2011). The bZIP transcription factor FD, a positive regulator of flowering, interacts with the flowering repressors BROTHER OF FT and TFL1 (BFT). This interaction interferes with the normal interaction between FT and FD, resulting in delayed flowering of *Arabidopsis* under salt stress (Ryu et al., 2014) (**Figure 1**). The osmotic response protein HOS1 ubiquitinates the transcription factor SPL9 under salt stress, promoting its degradation and thereby delaying flowering in *Arabidopsis* (Jiao et al., 2024) (**Figure 1**). In rice, components of the Evening Complex (EC), including OsELF4a, OsELF3-1, and OsLUX, play important roles in response to salt stress (**Table 1**). Under salt stress, these proteins bind to the promoter of *OsGI* (*GIGANTEA*), repress its expression, and delay flowering (Andrade et al., 2022). Most studies on flowering regulation under salt stress focus mainly on the model plant *Arabidopsis*, whereas studies on important crop species, such as rice, remain limited and are in the early stages. Therefore, further research on the mechanisms underlying the regulation of flowering under salt stress in economically important crops, such as rice, soybean, and maize, is highly important for the breeding of stress-resistant and early-maturing varieties.

### 2.4 High temperature-mediated regulation of flowering

Global warming is profoundly altering the flowering times of plant species worldwide. Failure to complete the floral transition at the optimal developmental stage compromises reproductive success and often results in yield reduction in crops. High temperature generally promotes flowering, primarily through the activation of flowering inducers-such as PIF4, miR172, and SPLs (Feng et al., 2021; Koini et al., 2009) and through the transcriptional suppression of flowering repressors, including miR156, SVP, and DELLA proteins. These regulatory inputs converge on FT, the central integrator of flowering signals, which mediates the temperature-responsive floral transition (**Figure 1**, **Table 2**). At the molecular level, the transcriptional regulation of *FT* involves FLOWERING LOCUS M (FLM) and SHORT VEGETATIVE PHASE (SVP). FLM promotes *FT* expression, whereas SVP acts as a repressor. Notably, FLM undergoes temperature-dependent alternative splicing, generating two isoforms: FLM-β and FLM-δ. Under high temperatures, FLM-δ becomes predominant and forms a SVP–FLM-δ complex, which interferes with the ability of SVP to repress *FT*, thereby accelerating flowering (Jin et al., 2022; Lee et al., 2013; Posé et al., 2013).

**Table 2:** Protein interactors of various flowering regulatory factor in plants.

Protein interaction	Molecular function	Ref.
FKF1-DELLA	Promotes the ubiquitination and degradation of DELLA proteins	(Murase et al., 2008)
GID1-SCFSLY1/GID2	Promotes expression of flowering genes	(Ueguchi-Tanaka et al., 2005)
DELLA-NF-Ys	Represses *SOC1* expression	(Fernández et al., 2016) (Hwang et al., 2019)
DELLA-MYC3	Promotes *MYC3* expression	(Bao et al., 2019)
FD-BFT	BFT compete for interaction with FD and antagonize FT activity	(Ryu et al., 2014)
HOS1-SPL9	Promotes the ubiquitination and degradation of SPL9	(Jiao et al., 2024)
OsELF4a/OsELF3-1/OsLUX-OsGI	Represses *OsGI* expression	(Andrade et al., 2022)
COI1-JAZ	Promotes *TOE1* and *TOE2* expression	(Browse and Wallis, 2019)
DELLA-JAZ	Promotes *TOE1* and *TOE2* expression	(Browse and Wallis, 2019)
SVP-FLM-o	Promotes *FT* expression	(Jin et al., 2022) (Lee et al., 2013) (Posé et al., 2013)
FLC-CO	Antagonistically regulate the expression of FT	(Kinmonth-Schultz et al., 2021)

Phytohormones also play critical roles in high temperature–induced flowering. Elevated temperatures upregulate the expression of GA biosynthesis genes *AtGA20ox1* and *AtGA3ox1*, resulting in increased accumulation of the bioactive gibberellin GA_4_ (Stavang et al., 2009; Yamaguchi, 2008). Binding of GA_4_ to its receptor GID1 facilitates recruitment of the SCF^SLY1/GID2 complex, which targets DELLA proteins—negative regulators of GA signaling—for ubiquitin-dependent degradation. DELLA removal releases their repression on flowering activators such as miR172, SPLs, and PIF4, thus promoting flowering under high temperature (Galvão et al., 2012; Sun, 2011; Van De Velde et al., 2017; Yu et al., 2012) (**Figure 1**). In addition, brassinosteroids (BRs) contribute to heat-responsive flowering regulation. High temperature induces nuclear accumulation of the BR signaling transcription factor BZR1, which directly binds to the *PIF4* promoter to enhance its expression (Ibañez et al., 2018). This activation further promotes *FT* transcription, collectively accelerating flowering under elevated temperatures.

## 3 Biotic stress and flowering regulation

Biotic stresses, including pathogens and pests, can profoundly influence flowering by altering the expression of key flowering genes (e.g., *FLC*, *FT*, *GI*), disrupting floral organogenesis (e.g., stigma, filament, anther), and impairing pollen viability (Lyons et al., 2015; Fan et al., 2022; Li et al., 2025) (**Table 1**).

Fungal pathogens exhibit strong tissue specificity in their infection strategies. Root-colonizing fungi such as *Piriformospora indica* and *Pochonia chlamydosporia* systemically accelerate flowering by manipulating phytohormonal pathways and directly regulating flowering gene expression (Cheng et al., 2004; Kim et al., 2017; Pan et al., 2017). In contrast, floral-infecting fungi such as *Ustilaginoidea virens* and *Claviceps purpurea* employ highly localized strategies, interfering with gametophyte development and seed formation through effector proteins and physical replacement of reproductive structures (Sun et al., 2020).

Viruses display distinct infection and transmission strategies. Unlike most pathogens, viruses are often excluded from meristematic tissues. However, species such as *PNRSV* and *ToBRFV* exploit pollen as transmission vectors, impairing pollen viability and tube growth (Amari et al., 2009; Avni et al., 2022). Owing to their systemic nature, viruses can also modulate flowering time by perturbing phytohormone signaling networks. In addition, biotic stress imposed by viral infection can induce early flowering. For example, the Foxtail Mosaic Virus–based VIF system (FoMViF) promotes early flowering in monocots and cereals, largely through upregulation of *FT*, although the role of DNA methylation in this process remains unclear. Moreover, small RNAs (sRNAs) have been shown to mediate methylation of both host and viral DNA, ultimately promoting precocious flowering in infected hosts (Zhang et al., 2015).

Insect herbivory further adds to the complexity of biotic stress–flowering interactions. In *Arabidopsis*, herbivore attack activates the jasmonic acid (JA) signaling pathway, which plays a dual role in defense and developmental regulation. JA accumulation promotes COI1-dependent degradation of JASMONATE-ZIM DOMAIN (JAZ) repressors, thereby releasing repression on TOE transcription factors. As a result, TOE1 and TOE2 more strongly suppress *FT*, leading to delayed flowering (Browse and Wallis, 2019; Li et al., 2004). In parallel, DELLA proteins physically interact with JAZ, alleviating JAZ-mediated inhibition of TOE activity and reinforcing *FT* repression, thus establishing a genetic link between the GA and JA pathways in the regulation of flowering (**Figure 1**, **Table 2**). Collectively, biotic stresses regulate flowering through diverse mechanisms—including transcriptional reprogramming, hormonal crosstalk, epigenetic modification, and reproductive organ disruption. These findings underscore the intricate balance plants must maintain between defense and reproduction when challenged by pathogens and herbivores.

## 4 Phytohormone-mediated regulation of flowering

### 4.1 Regulation of flowering by abscisic acid

Abscisic acid (ABA), a central stress-responsive phytohormone, restricts crop growth and development under adverse conditions while simultaneously modulating flowering time through multiple pathways across species. However, the role of ABA in floral transition remains debated, as it exerts both promotive and inhibitory effects depending on the environmental context. This functional divergence arises primarily from its differential regulatory actions under distinct photoperiods and across crop species.

**Figure 2:**
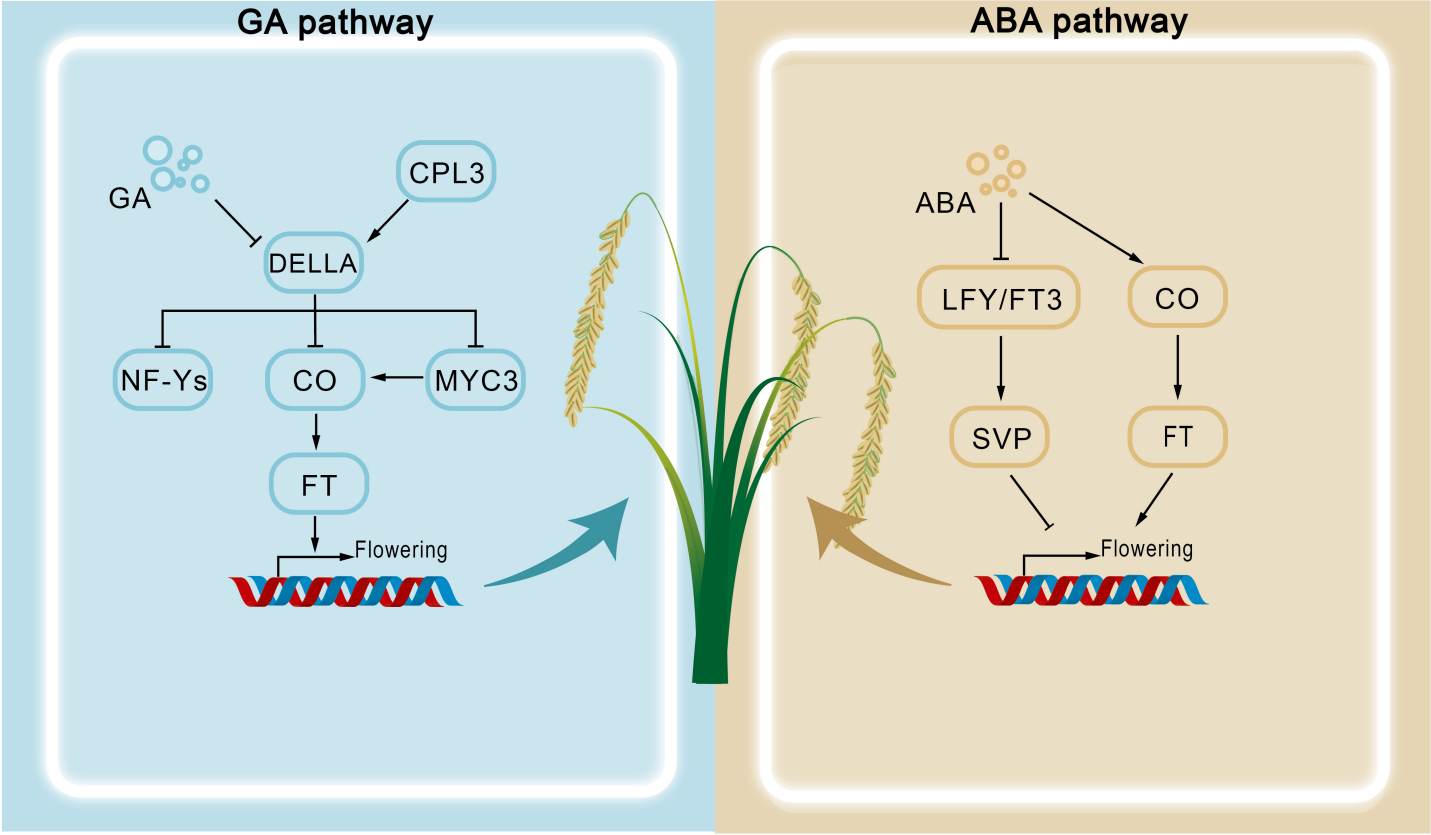
Molecular mechanisms of GA and ABA in regulating flowering time.

In *Arabidopsis*, ABA promotes early flowering under drought stress by interacting with components of the photoperiod pathway, such as GIGANTEA (GI) and CONSTANS (CO), thereby ensuring reproductive success. Specifically, ABA signaling enhances CO binding to the CORE cis-element of the *FLOWERING LOCUS T* (*FT*) promoter, inducing *FT* expression and accelerating flowering (Kinmonth-Schultz et al., 2021) (**Figure 2**). This ABA-induced pathway may be further coordinated by GI, although the precise molecular mechanism remains to be clarified (Robustelli Test et al., 2025). Supporting this view, mutants of ABA biosynthesis genes *ABA DEFICIENT 1* (*ABA1*) and *ABA2* exhibit delayed flowering under long-day (LD) conditions, but not under short-day (SD) conditions, highlighting ABA's promotive role under LD photoperiods (Riboni et al., 2013). Mechanistically, this ABA-dependent acceleration occurs largely through upregulation of *FT* and *TWIN SISTER OF FT* (*TSF*) during drought stress (Riboni et al., 2013, Riboni et al., 2016). By contrast, under SD conditions, drought stress delays flowering. This inhibitory effect is mediated by ABA-dependent upregulation of flowering repressors such as *SHORT VEGETATIVE PHASE* (*SVP*) (Wang et al., 2018), which suppress downstream floral identity genes (Riboni et al., 2013) (**Figure 2**). Consistently, loss-of-function mutants of ABA signaling components *ABI3*, *ABI4*, and *ABI5* display accelerated flowering, whereas their overexpression delays floral initiation (**Table 1**). These findings emphasize the complexity of ABA concentration–dependent regulation and pathway integration, which remain incompletely understood.

Importantly, ABA's role in flowering exhibits significant interspecies divergence. In *Crocus sativus*, for instance, exogenous ABA application inhibits flowering by repressing both floral induction and development, a striking contrast to the ABA-promoted drought escape mechanism in *Arabidopsis* (Singh et al., 2023). This suggests evolutionarily conserved yet species-specific regulatory frameworks. Accumulating evidence also highlights extensive hormonal crosstalk involving ABA. For example, ABI4 activates the gibberellin catabolic gene *GA2ox7*, reducing bioactive GA levels and thereby indirectly modulating flowering via GA homeostasis (Shu et al., 2016). Moreover, strigolactones (SLs) and ABA share a common biosynthetic precursor and demonstrate reciprocal regulation during abiotic stress responses. Under favorable growth conditions, ABA–SL equilibrium maintains developmental homeostasis, ensuring normal crop growth (Chi et al., 2021). In contrast, drought stress suppresses SLs while elevating ABA, activating defense pathways and accelerating flowering (Bai et al., 2024).

### 4.2 Regulation of flowering time by gibberellins

Gibberellins (GAs) are a major class of phytohormones that profoundly influence crop growth and development, particularly stem elongation and the floral transition. GA generally promotes flowering by targeting DELLA proteins—transcriptional repressors that constrain floral initiation—for degradation (Sun, 2011). In many crop species, GA functions as a key flowering promoter, especially in those requiring vernalization or LD photoperiods to induce floral development.

DELLA proteins serve as central repressors within GA signaling, with their activity tightly regulated at the cellular level. Under stress conditions, enhanced expression of GA biosynthetic enzymes GA20ox and GA3ox increases the accumulation of bioactive GA (Ito et al., 2018). Bioactive GA binds to its receptor GIBBERELLIN INSENSITIVE DWARF1 (GID1), inducing a conformational change that facilitates recruitment of the SCF^SLY1/GID2 E3 ubiquitin ligase complex. This interaction forms the GA–GID1–DELLA ternary complex, leading to ubiquitination and proteasomal degradation of DELLAs (Ueguchi-Tanaka et al., 2005; Murase et al., 2008). Depletion of DELLA proteins relieves transcriptional repression on flowering regulators, thereby accelerating flowering under LD conditions (Ito et al., 2018) (**Table 2**). Intriguingly, FKF1, a flowering regulator, promotes DELLA ubiquitination, while DELLAs suppress *FKF1* transcription, establishing a negative feedback loop that fine-tunes floral initiation (Yan et al., 2020).

Beyond protein degradation, GA also regulates the transcription of core flowering genes such as *FT* and *SOC1*. The nuclear factor Y (NF-Y) transcription complex integrates photoperiodic and GA signals to activate *SOC1* and *FT*. Under GA-deficient conditions, stabilized DELLAs sequester NF-Y subunits, repressing *SOC1* expression. GA-mediated DELLA degradation liberates NF-Y, enabling it to complex with CONSTANS (CO) and activate *SOC1* and *FT*, thus promoting flowering (Fernández et al., 2016; Hwang et al., 2019). In addition, DELLAs interact with the bHLH transcription factor MYC3, stabilizing it and allowing MYC3 to compete with CO for binding to the *FT* promoter. This antagonism impairs CO-mediated activation of *FT*, thereby delaying the floral transition (Bao et al., 2019) (**Figure 2**, **Table 2**).

The promotive effects of GA on flowering hold significant agronomic potential. For instance, exogenous GA application accelerates development and flowering in barley (*Hordeum vulgare* L.), whereas trinexapac-ethyl, a GA biosynthesis inhibitor, markedly delays flowering in a dose-dependent manner, independent of application timing (Kupke et al., 2021). Such vegetative phase extension through GA suppression enables strategic alignment of flowering with favorable reproductive windows, thereby maximizing yield potential. Conversely, GA promotion can be leveraged for precocious maturity where early flowering is desirable. These findings highlight GA modulation as a versatile agronomic strategy for optimizing crop productivity and adaptability.

## 5 Discussion

Floral transition at the appropriate developmental stage is critical for plant survival and plays a decisive role in improving crop yield. Yet, diverse biotic and abiotic stresses increasingly disrupt flowering schedules in many crops, thereby compromising productivity. This review synthesizes the molecular mechanisms underlying flowering regulation under the combined influences of photothermal cues, phytohormones, and environmental stresses, aiming to provide an integrative framework for understanding floral transition under adverse conditions and to inform strategies for crop improvement.

Despite recent advances, significant challenges remain in breeding stress-resilient cultivars. Flowering responses to stress are complex quantitative traits, governed by multiple genes (Atlin et al., 2017), and further complicated by the intricate cross-talk between stress signaling pathways and gene regulatory networks. This complexity hampers genetic dissection and hinders the precise identification of causal loci. Moreover, breeders face the persistent challenge of balancing stress tolerance with yield stability, not only within a single generation but also across successive breeding cycles. The simultaneous enhancement of stress resistance, flowering time optimization, and yield potential remains an elusive goal. Another pressing issue is the limited adaptability of many stress-resilient cultivars. Such genotypes often exhibit narrow ecological adaptation, resulting in significant performance variation across different geographical regions and production environments. This restricts their large-scale deployment and practical utility.

Looking forward, future efforts should emphasize the systematic integration of multi-omics approaches—including genomics, transcriptomics, proteomics, and metabolomics—to unravel the functional and mechanistic basis of gene clusters that govern stress resilience and flowering time. Building comprehensive gene regulatory network models will provide new insights into the dynamic interplay between environmental cues and developmental programs. Furthermore, leveraging big data analytics and artificial intelligence (AI) offers substantial promise for enhancing the precision and efficiency of breeding pipelines. By coupling multi-omics datasets with advanced computational tools, breeders can accelerate the development of cultivars that combine multi-stress resilience with superior agronomic performance, ultimately contributing to global food security under changing climates.

## Data Availability

The original contributions presented in the study are included in the article/supplementary material. Further inquiries can be directed to the corresponding authors.
